# Sociodemographic profile of medicines users in Brazil: results from the 2014 PNAUM survey

**DOI:** 10.1590/S1518-8787.2016050006119

**Published:** 2016-12-01

**Authors:** Andréa Dâmaso Bertoldi, Tatiane da Silva Dal Pizzol, Luiz Roberto Ramos, Sotero Serrate Mengue, Vera Lucia Luiza, Noemia Urruth Leão Tavares, Mareni Rocha Farias, Maria Auxiliadora Oliveira, Paulo Sergio Dourado Arrais

**Affiliations:** I Departamento de Medicina Social. Faculdade de Medicina. Universidade Federal de Pelotas. Pelotas, RS, Brasil; II Departamento de Produção e Controle de Medicamentos. Faculdade de Farmácia. Universidade Federal do Rio Grande do Sul. Porto Alegre, RS, Brasil; III Departamento de Medicina Preventiva. Escola Paulista de Medicina. Universidade Federal de São Paulo. São Paulo, SP, Brasil; IV Programa de Pós-Graduação em Epidemiologia. Faculdade de Medicina. Universidade Federal do Rio Grande do Sul. Porto Alegre, RS, Brasil; V Departamento de Política de Medicamentos e Assistência Farmacêutica. Escola Nacional de Saúde Pública Sérgio Arouca. Fundação Oswaldo Cruz. Rio de Janeiro, RJ, Brasil; VI Departamento de Farmácia. Faculdade de Ciências da Saúde. Universidade de Brasília. Brasília, DF, Brasil; VII Departamento de Ciências Farmacêuticas. Centro de Ciências da Saúde. Universidade Federal de Santa Catarina. Florianópolis, SC, Brasil; VIII Departamento de Farmácia. Faculdade de Farmácia, Odontologia e Enfermagem. Universidade Federal do Ceará. Fortaleza, CE, Brasil

**Keywords:** Drug Utilization, Socioeconomic Factors, Health Inequalities, Health Surveys

## Abstract

**OBJECTIVE:**

To analyze the prevalence of medicine use by the Brazilian population and its distribution according to sociodemographic factors.

**METHODS:**

Study using data from the *Pesquisa Nacional de Acesso, Utilização e Promoção do Uso Racional de Medicamentos* (PNAUM – National Survey on Access, Use and Promotion of Rational Use of Medicines), a nationwide household survey of a representative sample of the Brazilian urban population. The data were collected between September 2013 and February 2014. The overall use of medicines, defined as the use of any medicine, use of medicines for treating chronic medical conditions and for acute health conditions, was evaluated. The independent variables included gender, age group, socioeconomic position, and region of Brazil. Analyzes included prevalence calculations, 95% confidence intervals (95%CI) and Pearson Chi-square tests to evaluate the differences between groups, considering a 5% level of significance.

**RESULTS:**

The prevalence of medicines use was 50.7% (95%CI 49.3–52.2), with 39.3% (95%CI 37.5–41.1) accounting for men and 61.0% (95%CI 59.3–62.6) for women. Medicines use was observed to increase with increasing age, except among children within the zero to four years age group. The lowest prevalence for medicines use was found among those with a low socioeconomic position and those who reside in the North region of Brazil. The prevalence of medicine use to treat chronic diseases was 24.3% (95%CI 23.3–25.4), whereas it was 33.7% (95%CI 32.1–35.4) for treating acute diseases.

**CONCLUSIONS:**

We found extensive variability in the prevalence of medicines use across regions of Brazil. The poorest regions (North, Northeast, and Midwest) have a lower prevalence of medicines use to treat chronic diseases, indicating the need to minimize inequalities in access to medicines within the country.

## RESUMO

**OBJETIVO:**

Analisar a prevalência do uso de medicamentos pela população brasileira e sua distribuição segundo aspectos sociodemográficos.

**MÉTODOS:**

Estudo com dados da Pesquisa Nacional de Acesso, Utilização e Promoção do Uso Racional de Medicamentos (PNAUM), pesquisa nacional de base populacional, realizada entre setembro de 2013 e fevereiro de 2014 em residências de municípios urbanos. Avaliou-se o uso de todos os medicamentos (global), uso de medicamentos para doenças crônicas e uso de medicamentos para doenças agudas. As variáveis independentes utilizadas foram sexo, idade, classificação econômica e região do País. Foram calculados prevalências e intervalos de confiança de 95% (IC95%) e aplicado teste Qui-quadrado de Pearson para avaliação das diferenças entre os grupos, considerando o nível de significância de 5%.

**RESULTADOS:**

A prevalência global de uso de medicamentos foi de 50,7% (IC95% 49,3–52,2), sendo 39,3% (IC95% 37,5–41,1) no sexo masculino e 61,0% (IC95% 59,3–62,6) no sexo feminino. Observou-se aumento nas prevalências de uso com a idade (exceto de zero a quatro anos). As menores prevalências de uso ocorreram no grupo mais pobre e na região Norte do País. A prevalência de uso de medicamentos para doenças crônicas foi de 24,3% (IC95% 23,3–25,4) e para doenças agudas foi de 33,7% (IC95% 32,1–35,4).

**CONCLUSÕES:**

Existe grande variabilidade nas prevalências globais de uso de medicamentos por regiões brasileiras. As regiões consideradas mais pobres (Norte, Nordeste e Centro-Oeste) apresentam menores prevalências de uso de medicamentos para doenças crônicas, o que indica a necessidade de minimizar as desigualdades no acesso aos medicamentos dentro do País.

## INTRODUCTION

The last century has seen medicines become highly relevant social assets to humanity as a whole, with them contributing towards improved quality of life and increased life expectancy for people^2^. It is reasonable to consider medicines as an indispensable resource for most therapeutic plans^16^.

There are important differences in determining the profile of medicine use when evaluating samples from institutions (hospitals, primary healthcare units, schools), samples from sick individuals and population-based samples^5^. Population-based samples can provide a true picture of the population medicine use profile; this scenario contributes to estimate the need for medicines as health resources, which is essential information for directing public policy and new research in the area^3^ ^,^ ^5^ ^,^ ^25^.

There have been international studies to evaluate medicines use that showed the overall prevalence of use (any medicine) ranging from 49.6% (Cuba) to 74.7% (Germany). The prevalence of medicine use in two Spanish communities were found to be 65.1% and 67.0%, respectively, utilization being higher among women and increasing in frequency along with age^9^ ^,^ ^22^.

In Brazil, studies on medicine utilization found wide variations in prevalence, with values raging between 49.0% and 56.9%. These results were identified respectively by Carvalho et al.^10^, among individuals aged 18 years or more, and by a study developed by the *Conselho Nacional de Secretários de Saúde (*Brazilian National Council of Health Secretaries)^11^, among individuals aged 16 years or more. The latter study found that the prevalence of use in different geographic regions in Brazil was highest in the South (62.3%) and Southeast (62.2%), followed by the Midwest (50.5%), Northeast (50.2%) and North regions (43.1%).

Other studies carried out in Brazilian cities and the Federal District, using different recall periods and age groups, showed a high variability in the prevalence of medicines use by the populations under investigation (35.7% to 76.5%). In Brasilia (FD), the prevalence was 35.7%^14^, in Florianopolis (SC), 76.5%^8^, in Campinas (SP), 48.5%^12^, in Fortaleza (CE), 49.7%^2^, and in Pelotas (RS), 65.9%^4^. Medicines use was higher among women and increases according to age and socioeconomic status during the studies conducted in Campinas, Fortaleza and Pelotas^2^ ^,^ ^4^ ^,^ ^12^.

In-depth health surveys have not yet been conducted in Brazil to evaluate medicines use, due to the fact that the extensive detail the theme requires would absorb much of the research itself. *Pesquisa Nacional de Acesso, Utilização e Promoção do Uso Racional de Medicamentos* (PNAUM – National Survey on Access, Use and Promotion of Rational Use of Medicines) was specifically designed to investigate the use of medicines in a representative sample of the Brazilian population.

The purpose of this study was to analyze the prevalence of medicines use in Brazilian geographical regions, considering the use of any medicine and those used specifically for acute or chronic diseases, according to sociodemographic characteristics of the population.

## METHODS

The PNAUM was a population-based cross-sectional study, with the data being collected between September 2013 and February 2014 in households in Brazilian cities. Face-to-face interviews were conducted in these households, and the data were recorded using tablets and software that had been specifically developed for applying questionnaires.

The study population was made up of residents permanently residing in private households within the Brazilian territory’s urban area, and included individuals from all age groups. Sample size calculations considered eight demographic aspects (different genders and ages) that were repeated in each of Brazil’s major geographic regions, resulting in 40 sampling domains. A sample size of 960 interviews per sampling domain was the result, totaling 38,400 interviews. The sample was selected based on three stages: municipality (primary unit), census sector and household. Participants were selected from households, based on the expected proportion of each age group and gender to compose the final sample. The sampling process was complex and resulted in a sample that ensured national representation for the five Brazilian regions stratified by sex and age groups. Details regarding the sampling and data collection logistics are described in the PNAUM methodological article^18^.

The questionnaires were developed by researchers from seven universities in Brazil, having been standardized and tested previous to their implementation.

Medicine use was investigated based on three perspectives. The first sought to find information regarding the current use of medicines for chronic diseases. This involved information on previous diagnosis and medical recommendation to use medicines to treat specific chronic diseases (hypertension, diabetes, heart disease, high cholesterol, a history of stroke, chronic respiratory diseases, arthritis, arthrosis or rheumatism, and depression). Other chronic diseases lasting more than six months were also investigated.

The second perspective aimed to evaluate medicines use for any possible health problems, generally characterized by signs, symptoms and acute conditions treated with medicines. Investigating these cases involved inquiring into medicines use 15 days prior to the research for specific health issues or groups of medicines (infection, sleeping medicines, nerves, stomach or bowel problems, pain, fever, flu, colds or allergic rhinitis, vitamin, mineral supplement, appetite stimulant or tonic) and any other medicines that had been unreported.

The third perspective separately investigated current contraceptive use, depending on the characteristics of this group of medicines that is unrelated to disease and has everything to do with contraception. The complete questionnaire can be seen on the PNAUM survey website (http://www.ufrgs.br/pnaum).

The analyzed outcomes were as follows: 1) overall prevalence of medicines use (use of at least one medicine for chronic disease, eventual or contraceptive use); 2) prevalence of medicines use for chronic diseases (use of at least one medicine for chronic diseases); 3) prevalence of medicines use for eventual or acute diseases (use of at least one medicine to treat acute signs, symptoms or conditions).

The independent variables under analysis were: age group (0-4; 5-9; 10-19; 20-29 30-39; 40;-49; 50-59 60-69; 70;-79; 80 years old or more); economic classification (A/B; C; D; E), according to the Brazilian Economic Classification Criterion of the *Associação Brasileira de Empresas de Pesquisa* (CCEB 2013/ABEP – http://www.abep.org/), and residential geographical region (North; Northeast; Southeast; South, Midwest).

Analyses for this article were performed using the total sample and the sample stratified by gender. All analyses were conducted with Stata version 12 (StataCorp LP, College Station, Texas, EUA) statistical software, using the set of svy commands designed to analyze complex samples. The analyses guaranteed the required weighting by contemplating the characteristics of the sample design that used different sampling fractions and post-stratification weights to correct the response rate flaw. The calculated percentages were weighted to adjust the PNAUM sample’s demographic distribution to the Brazil’s population.

In addition to the prevalence estimates, 95% confidence intervals (95%CI) were calculated and the Pearson’s Chi-square test was applied to assess the statistical significance of the differences among the groups, considering a 5% level of significance.

The study’s design was submitted and approved by the *Comissão Nacional de Ética em Pesquisa* (CONEP – Brazilian National Commission for Ethics in Research). All the interviews were conducted after the respondent or his/her legal guardian (in the case of individuals under 18 years old or those unable to answer the questions themselves) had signed a free prior and informed consent term.

## RESULTS

The study sample included 41,433 individuals with a distribution that was comparable with that from the 2010 Brazilian Population Census (percentages weighted by sampling weights). The response rates from households were around 50.0%, which includes the losses from the households that were not visited. The response rates from the individuals were around 90.0%. The methodological article includes a detailed table of the response rates by gender and age group as well as by geographical region^18^.

The weighted estimates indicated that 52.8% of the respondents were female, 22.3% of whom belonged to economic classes A or B, 57.1% were adults aged between 20 and 59 years, and with 45.9% residing in the Southeast region of Brazil (Table 1).

**Table 1:** Distribution of the sample and prevalencea of medicines use in Brazil, according to sociodemographic characteristics. PNAUM, Brazil, 2014. (N = 41,433)

Sociodemographic characteristic	Sample	Prevalence of medicines use					

		All		Male		Female	
			
	%	Prevalence	95%CI	Prevalence	95%CI	Prevalence	95%CI
Age group^b^		< 0.001^c^		< 0.001		< 0.001	
0-4	6.2	42.0	39.5–44.6	42.6	39.5–45.7	41.4	38.6–44.3
5-9	7.5	25.3	22.0–28.6	26.7	21.6–31.8	23.9	19.4–28.5
10-19	16.0	30.6	27.9–33.3	24.5	20.7–28.2	36.5	32.7–40.4
20-29	16.5	48.0	45.4–50.6	31.4	26.8–35.9	64.4	62.0–66.8
30-39	15.3	50.6	47.9–53.2	34.8	30.3–39.3	64.5	61.8–67.3
40-49	13.8	54.1	52.0–56.2	39.0	36.6–41.4	67.5	65.3–69.7
50-59	11.5	64.7	62.5–66.8	50.3	47.0–53.5	76.1	74.0–78.2
60-69	6.9	77.0	75.3–78.7	68.3	65.7–70.9	83.8	81.9–85.6
70-79	4.3	85.8	84.0–87.5	79.8	76.1–83.4	89.9	88.2–91.6
≥ 80	2.0	88.6	86.1–91.1	85.2	81.6–88.9	90.9	87.6–94.2
Economic classification^d^		0.027		0.423		0.015	
A/B	22.3	51.9	49.2–54.5	40.7	37.0–44.4	62.5	59.9–65.1
C	55.3	51.1	49.4–52.8	39.2	37.2–41.2	61.5	59.5–63.5
D	17.7	50.1	47.5–52.8	39.1	35.7–42.6	59.2	56.2–62.2
E	4.6	43.5	38.2–48.8	34.6	27.6–41.6	53.2	47.0–59.3
Region		0.001		0.057		< 0.001	
North	7.5	42.4	38.0–46.7	35.7	31.7–39.6	48.6	43.1–54.1
Northeast	24.3	53.4	51.2–55.7	42.7	39.7–45.6	62.6	60.1–65.0
Southeast	45.9	50.8	48.1–53.5	39.1	35.7–42.4	61.3	58.3–64.3
South	14.3	49.8	46.8–52.9	36.5	32.9–40.1	61.8	59.0–64.6
Midwest	7.9	51.8	49.2–54.4	39.5	36.2–42.7	63.9	60.7–67.0

Total	100	50.7	49.3–52.2	39.3	37.5–41.1	61.0	59.3–62.6

^a^ Percentages adjusted by sample weights and post-stratification according to age and gender. Weighted sample distribution by gender: 47.2% (male) and 52.8% (female).

^b^ In years.

^c^ Pearson’s Chi-square test.

^d^ The economic classification variable according to the Brazilian Economic Classification Criterion of the *Associação Brasileira de Empresas de Pesquisa* (http://www.abep.org) had 77 ‘*missing’*.

**Figure 1:**
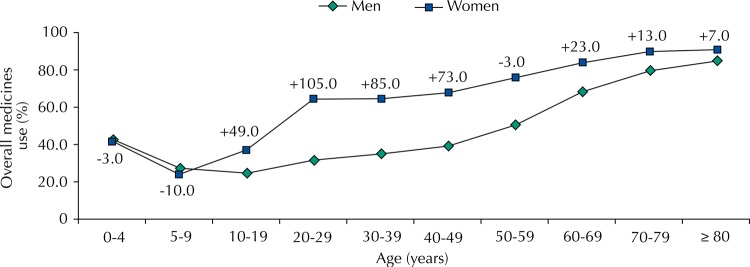
Prevalence of overall medicines use in Brazil according to gender and age group. The values represent the percentage difference in medicines use by women compared to men. PNAUM, Brazil, 2014.

The overall prevalence of medicines use was 50.7% (95%CI 49.3–52.2), with 39.3% (95%CI 37.5–41.1) being males and 61.0% (95%CI 59.3–62.6) females. After the sample was stratified by age groups, there was an observed increase in the prevalence of use along with increasing age, the exception being the first age group (zero to four years), which is about 70.0% higher than the following group (five to nine years). The largest use of medicines by females begins from the 10-19 years age group (Table 1). Figure 1 shows the extent of the differences for each age group. Women use more than twice as many medicines as men between the ages of 20 and 29.

The smallest prevalence of use was in the group with the smallest economic classification (class E) in both gender groups. The groups with the highest purchasing power used about 20.0% more medicines than those with the lowest economic classification (Table 1).

The geographical region which had highest prevalence of medicines use was the Northeast (male) and their prevalence was statistically higher than those observed in the North. The lowest prevalence occurred in the North region and was statistically lower than those observed in the other regions in the female gender (Table 1).

Table 2 shows the results related to the treatment of chronic diseases. The prevalence of medicines use for chronic diseases was 24.3% (95%CI 23.3–25.4): 18.7% (95%CI 17.6–19.9) for males and 29.4% (95%CI 28.1–30.7) for females. Considering the total sample, the prevalence increased with age – the oldest age group (80 years or more) used 14 times more medicines than the youngest age group (0-4 years). The prevalence increased from the 20 to 29 years age group among males, while the growth begins in the 10 to 19 years age group among females.

**Table 2:** Prevalencea of medicines use to treat chronic diseases in Brazil, according to sociodemographic characteristics. PNAUM, Brazil, 2014. (N = 41,433)

Sociodemographic characteristic	Prevalence of medicines use to treat chronic diseases					

	All		Male		Female	
		
	Prevalence	95%CI	Prevalence	95%CI	Prevalence	95%CI
Age group^b^	< 0.001^c^		< 0.001		< 0.001	
0-4	5.7	4.8–6.5	6.4	5.2–7.5	5.0	3.8–6.1
5-9	5.2	3.6–6.8	6.0	3.4–8.5	4.5	2.3–6.6
10-19	5.3	4.1–6.5	4.9	3.2–6.5	5.7	4.0–7.4
20-29	7.5	6.1–8.9	5.8	3.7–7.9	9.2	7.5–10.8
30-39	14.9	13.2–16.5	11.2	8.5–13.9	18.1	16.2–19.9
40-49	28.9	27.4–30.4	20.5	18.7–22.4	36.4	34.3–38.5
50-59	49.9	48.0–51.8	36.4	33.7–39.1	60.5	58.0–63.0
60-69	66.2	64.2–68.1	56.7	53.7–59.7	73.5	71.3–75.7
70-79	78.5	76.4–80.6	71.6	67.8–75.5	83.2	81.1–85.4
≥ 80	79.7	75.6–83.8	73.4	70.7–80.0	82.7	76.7–88.6
Economic classification^d^	0.011		0.033		0.038	
A/B	26.5	24.6–28.4	21.0	18.6–23.4	31.8	29.6–34.1
C	24.2	23.0–25.4	18.6	17.2–20.0	29.1	27.6–30.6
D	22.4	20.6–24.3	16.4	14.4–18.4	27.4	24.9–29.9
E	23.1	19.6–26.6	18.2	13.7–22.6	28.5	23.8–33.3
Region	< 0.001		< 0.001		< 0.001	
North	14.0	12.2–15.7	11.6	9.7–13.5	16.1	14.0–18.3
Northeast	20.5	18.9–22.1	15.4	13.6–17.1	24.8	22.7–27.0
Southeast	27.5	25.7–29.4	21.4	19.3–23.5	33.0	30.8–35.3
South	26.7	24.5–28.9	19.9	17.6–22.3	32.8	30.4–35.3
Midwest	23.2	21.4–25.1	18.0	15.8–20.2	28.4	26.4–30.3
Total	24.3	23.3–25.4	18.7	17.6–19.9	29.4	28.1–30.7

^a^ Percentages adjusted by sample weights and post-stratification according to age and gender.

^b^ In years.

^c^ Pearson’s Chi-square test.

^d^ The economic classification variable according to the Brazilian Economic Classification Criterion of the *Associação Brasileira de Empresas de Pesquisa* (http://www.abep.org) had 77 ‘missing’.

In regards to economic classification, the highest prevalence for medicines use was found in groups with the highest economic classification (A/B), and differences in which were statistically significant in relation to economic classification D in males. The prevalence of medicines use for treating chronic diseases was slightly higher in class E (poorest group) compared with class D (no statistical differences). The highest prevalence of medicines use for chronic diseases were found in the Southeast and South, with the entire sample showing significant statistical differences compared with the North and Northeast regions (Table 2).

Table 3 only shows the medicines used to treat acute health problems, which characterize eventual use of medicines. The prevalence of medicines use for these health problems was 33.7% (95%CI 32.1–35.4): 27.2% (95%CI 25.4–29.0) for males and 39.6% (95%CI 37.6–41.5) for females. The usage patterns for the total sample indicate an increasing trend from the five to nine years age group up to the 80 years or more age group. In the zero to four years age group, the prevalence of use of eventual medicines was the same as the adults above 30 years age group. The oldest age group (80 years or more) in males used two times more eventual medicines than the children in the five to nine years age group; this ratio was 2.5 times higher in females using the same comparison basis. There were no differences found in the use of such medicines by economic classification, despite their prevalence being approximately 20.0% higher in class A/B compared with class E (Table 3).

**Table 3:** Prevalencea of medicines use to treat acute diseases in Brazil, according to sociodemographic characteristics. PNAUM, Brazil, 2014. (N = 41,433)

Variable	Prevalence of medicines use to treat acute diseases					

	All		Male		Female	
		
	Prevalence	95%CI	Prevalence	95%CI	Prevalence	95%CI
Age group^b^	< 0.001^c^		< 0.001		< 0.001	
0-4	39.0	36.5–41.6	39.4	36.3–42.5	38.6	35.9–41.4
5-9	21.8	18.5–25.0	21.8	17.9–27.7	20.8	16.5–25.1
10-19	24.6	22.0–27.1	20.9	17.2–24.5	28.1	24.4–31.8
20-29	32.3	29.4–35.3	26.9	22.6–31.3	37.7	34.6–40.7
30-39	35.3	32.5–38.1	27.9	23.6–32.1	41.8	38.9–44.7
40-49	36.2	33.8–38.5	25.6	23.3–27.9	45.6	42.8–48.4
50-59	37.6	35.0–40.1	25.5	22.4–28.6	47.2	44.0–50.3
60-69	41.1	38.5–43.8	33.3	30.1–36.4	47.2	44.0–50.5
70-79	44.2	41.3–47.1	33.4	29.2–37.6	51.6	48.1–55.1
≥ 80	48.3	44.2–52.5	44.5	38.5–50.5	51.0	46.0–55.9
Economic classification^d^	0.097		0.314		0.263	
A/B	34.0	31.3–36.6	27.7	24.2–31.1	40.0	36.9–43.0
C	33.8	31.9–35.7	27.0	24.9–29.0	39.8	37.6–42.0
D	34.7	31.9–37.5	28.8	25.1–32.5	39.6	36.6–42.6
E	28.1	23.9–32.3	22.3	16.8–27.9	34.3	29.6–39.0
Region	< 0.001		< 0.001		< 0.001	
North	31.6	27.6–35.6	27.5	24.0–31.0	35.4	30.2–40.6
Northeast	40.2	37.8–42.7	33.6	30.4–36.7	45.9	43.3–48.5
Southeast	31.7	28.6–34.8	25.0	21.8–28.2	37.7	34.0–41.5
South	29.4	26.8–32.1	23.2	20.2–26.1	35.1	32.4–37.7
Midwest	35.4	32.6–38.2	28.3	24.8–31.7	42.4	39.2–45.7
Total	33.7	32.1–35.4	27.2	25.4–29.0	39.6	37.6–41.5

^a^ Percentages adjusted by sample weights and post-stratification according to age and gender.

^b^ In years.

^c^ Pearson’s Chi-square test.

^d^ The economic classification variable according to the Brazilian Economic Classification Criterion of the *Associação Brasileira de Empresas de Pesquisa* (http://www.abep.org) had 77 ‘missing’.

In regards to the geographical regions, the highest prevalence of medicines use was in the Northeast (with a statistically significant difference compared with the North, East and South) and the lowest in the South (with a statistically significant difference compared with the Northeast and Midwest regions) (Table 3).

**Figure 2:**
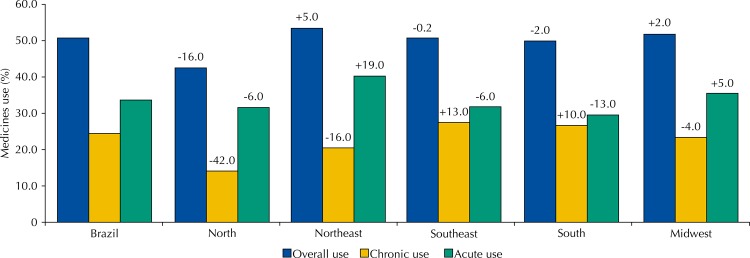
Prevalence of medicines use according to the type of use in the different regions of Brazil. The values represent the percentage difference between the prevalence of use in each region compared with the prevalence in Brazil. PNAUM, Brazil, 2014.

Figure 2 shows a comparison between the prevalence of overall usage, use of medicines for chronic diseases and use of medicines for acute conditions of each region in relation to Brazil as a whole. The North region had the most significant percentage differences in regards to overall use (-16.0%) and medicines for chronic diseases (-42.0%), these being smaller than the prevalence for such in Brazil. The Northeast region had the highest percentage for eventual medicines use (+19.0%), being greater than the national prevalence.

## DISCUSSION

Studies on medicines use can be conducted using routine or survey databases. Developed countries usually integrate data on medicines prescription and distribution, which generates databases that include information regarding the patient, medicines recommendation, dosage etc^23^. In Brazil, we have only consolidated data from industry based on retail sales. Thus, surveys are conducted with the objective of obtaining data about who used medicines, what was used and whether or not it was possible to get the necessary medicines.

The PNAUM survey was the first national study in Brazil that was specifically designed to evaluate medicines use in the five geographic regions of Brazil, which resulted in detailed information on all medicines used to treat chronic diseases, acute health situations and contraceptives. Among the Brazilian studies that included questions regarding medicines, the following are highlighted: the *Pesquisa Nacional por Amostra de Domicílios* (PNAD – National Household Sample Survey – http://www.ibge.gov.br), which records the continuous medicines use related to the last time the health services were used; the *Pesquisa de Orçamentos Familiares* (POF – Brazilian Household Budget Survey – http://www.ibge.gov.br) that records expenditure on medicines obtained over the last 30 days, purchase location and payment type; *Pesquisa Nacional de Demografia e Saúde* (PNDS *–* Brazilian National Demographic and Health Survey – http://www.ibge.gov.br); and the World Health Survey (WHS)^24^. The latter two used a similar strategy, in which they ask about prescription, use, sources to obtain medicines, primarily based on a list of chronic diseases.

Considering the overall prevalence of any type of medicines, slightly more than half the population used some kind of medicines within the periods investigated during the research, the results showing that use by women was 55.0% greater than that of men. The overall prevalence estimate is in line with other studies, which is true even when those with a smaller sample size are considered^2^ ^,^ ^4^ ^,^ ^10^ ^,^ ^12^ ^,^ ^14^. The greater use of medicines by women is also described in various national and international studies^20^. This difference is attributed to gender, in addition to the exclusive use of contraceptives, to factors related to increased use of health services and health care^21^, which begin during adolescence, due to complications related to the menstrual cycle and pregnancy. This is a period in which the differences start and continue to grow until the end of adulthood. Among the elderly, women are still the greatest medicines users, but the differences are smaller, a fact that is consistent with the literature^13^ ^,^ ^20^.

Medicines use by age group reflects the population’s profile of morbidities, starting with a high percentage for the use of eventual medicines in early childhood followed by a reduction and subsequent gradual increase along with increasing age, and with the percentage of medicines use to treat chronic diseases. The prevalence of medicines use is close to 90.0% in the 80 years or more age group. This result is in line with those from other studies, which indicate the same trend^4^ and show the impact of demographic and epidemiological transition in the use of medicines by the Brazilian population. In light of this scenario, efforts in terms of pharmaceutical service policy should be directed towards care strategies to guide these patients within the health care system, the aim being to guarantee access to essential medicines, to promote their rational use and to improve adherence to treatment. This study showed a higher medicines use by people belonging to the highest economic classification (A/B). Data from the literature consistently indicate this same result^2^ ^,^ ^4^ ^,^ ^10^ ^,^ ^12^, since medicines use depends on their access and this can be conditioned by the purchasing power of individuals when drugs are not available free of charge^7^.

We found a wide variability in the overall prevalence of medicines use in the different regions in Brazil. The lowest prevalence of medicines use is found in the North region, which is probably due to greater dependence and inefficiency of the public system regarding access to medicines, less medical diagnoses as a result of difficulties in accessing medical services, and even because of there likely being fewer locations to obtain medicines in the private sector than in the other Brazilian regions^1^. These variations are even sharper when the medicines are stratified by disease groups. According to the Brazilian Institute of Geography and Statistics (IBGE), the poorest regions (North, Northeast and Midwest) have the lowest prevalence of medicines use for chronic diseases compared with Brazil as a whole. On the other hand, the South and Southeast regions have the greatest prevalence of medicines use for these diseases. Being able to use medicines to treat chronic diseases can be considered a proxy of quality health care, since these diseases depend on a previous diagnosis for its control and evolution with the use of medicines^17^. At the same time, the Northeast and Midwest regions have the largest percentages of medicines use for eventual or acute diseases, which may be associated with self-medication and the pursuit of resolving acute health problems caused by medicines use^6^.

When we only consider treating chronic illnesses, the prevalence of medicines use is observed to be less than 10.0% up until 29 years of age, regardless of gender. From 30 years of age onwards, we found an increase in medicines use for these diseases. When we consider the entire population, the oldest age group (80 years or more) uses 5.3 times more medicines than the 30-39 years age group. When we only consider the male gender, this increase is 6.6 times, and only 4.6 times for women. This behavior based on age has already been covered in the literature^19^.

In relation to the treatment of acute diseases, the prevalence is high from childhood onwards, which is true for both boys and girls. The increases in the prevalence for males are 1.95 times greater in the 80 years or more age group compared with the five to nine years age group. This increase is 2.5 times higher for the female gender, indicating a greater stability in the use prevalence of these medicines compared with those used to treat chronic diseases.

It is possible to highlight the national and regional representation as a strong point of this study, which has allowed an overview of the overall use of medicines in Brazil to be created for the first time. Comparisons made by gender, age and economic class showed similar results regarding medicines use between the PNAUM survey and previous studies, and also built upon the differential characteristics observed in the patterns for medicines use for chronic and acute diseases in Brazil’s geographical regions.

At the same time, it is important to highlight that there are some inherent limitations to a survey of such magnitude. As the information depends on reports from respondents or their legal guardians, in the case of individuals aged less than 18 years old or persons unable to answer their own questionnaires, it is possible the existence of some degree of response error regarding which medicines were used. The periods adopted to investigate medicines use should also be considered. Current use was investigated for continuous use of medicines and contraceptives, and a 15-day recall period was used for medicines used to treat eventual health issues. Despite this procedure not being unusual in the literature^5^, comparisons with other studies should always consider that the prevalence may vary according to the investigation period for medicines use^5^.

Evaluating the Brazilian population’s profile for medicines use by age groups, gender and medicines type (for acute diseases or chronic diseases) made it possible to characterize the differences between the groups and tendencies among the geographic regions of Brazil.

The Brazilian Government has invested in strategies to improve access to medicines and enhance the quality of pharmaceutical services with initiatives such as regulating generic medicines (Law 9,787, February 10th, 1999), the Brazilian *Farmácia Popular* (Popular Pharmacy Program) (Decree 5,090, May 20th, 2004), the *QualifarSUS* (Ordinance GM/MS 1,214), among others. We hope that the results from this study can help guide the Brazilian government in regards to pharmaceutical services, the aim being to further improve the population’s access to essential medicines and ensure their rational use. The areas that were identified to have a lesser prevalence of medicines use to treat chronic diseases, such as the North and Northeast regions, should be prioritized to minimize the inequalities in terms of access to medicines.
